# Supplementary material for: Learning curve and surgical outcome of robotic assisted colorectal surgery with ERAS program
Source: Sci Rep. 2022 Nov 29;12:20566. doi: 10.1038/s41598-022-24665-w (PMC9709162; doi:10.1038/s41598-022-24665-w)
Supplement: Supplementary file 1 — Supplementary Information 1. [file 41598_2022_24665_MOESM1_ESM.zip › LSC Raw data-Ñ[▒K/083/H_ñw╝╨░O▒Kñσ.pdf]

正式摘要(電子簽章：000000196551781)		
W92 / W92 ☐ 英文標題(注意:無英文姓名-請補病人基本檔!)	☐【讀取】憑證	

臺中榮民總醫院

出院病歷摘要

轉入號： 轉出號：

[redacted]

(8)流水編號	
(9)入院日期	109/07/08 (2020) 直腸外科 W92 - 33
(10)轉科日期	
(11)出院日期	109/07/14 (2020) 住院天數計 6 日

診斷

- 入院：Prolapse of rectum
- 出院：Rectal prolaspe, s/p laparoscopic Anterior resection + rectopexy on 20200709.

主訴

- rectal prolapsed

病史

- This 22 years old male patient denied special past history. He noticed that there were intestines protruding out about 3 cm while defecation. He pulled them back but the symptom happened again during defecation. He denied any other GI discomfort or changed bowel habbit. Thus, he was admitted for operation.

體檢發現

- 2020/06/16 nasal mucosal pale , nasal allergy
  - General appearance :
    - easy looking , conscious clear , without cardiopulmonary distress
    - Vital signs自動帶入date 體溫(BT) 脈搏(P) 呼吸(R) 血壓(BP) 身高(BH) 體重(BW) BMI
      20200708 36℃ 55次/分 18次/分 111/72mmHg 169.9cm 71.7kg 24.8
    - Vital signs: B.P. 111/72 mmHg , P.R. 55 /min , R.R. 18 /min , B.T. 36 ℃
  - HEENT :
    - Eyes: no anemic conjunctiva, no icteric sclera, normal EOM
    - ENT: no otorrhea, no rhinorrhea, no erythematous mucosa, no injected tonsil, no deformity
  - Neck: supple, freely movable, trachea midline,no deformity, no engorged jugular vein , no palpable thyroid or lymph node enlargement
  - Chest:
    - Inspection: Grossly normal, symmetric and fully expansion, no accessory muscle used, no operation scar, no spider angiomata, no gynecomastia
    - Palpation: no palpable mass or nodule, no subcutaneous emphysema, no tenderness
    - Ausculation: clear breathing sound
  - Heart:
    - Percussion: normal heart size
    - Ausculation: regular heart beat, normal S1, S2, no gallop, no murmur
  - Abdomen:
    - Inspection: in
      - flat contour, symetrically, no scar, no stria, no dilated veins, no rashes
    - Ausculation: normoactive bowel sound, no abdominal bruits
    - palpation: soft, no muscle guarding or rigidity, no local tenderness, no rebounding pain, no palpable mass, no palpable pulsation
    - Percussion: no shifting dulllness, no hepatomegaly, no splenomagaly
  -

Peripheral pulsation :	CA	BA	RA	FA	PA	PTA	DPA

Right side :	++	++	++	++	++	++	++/+++
Left side :	++	++	++	++	++	++	++/+++

- Genito-anus: no tenderness or palpable mass lesion or rectal shelf, normal external genitalia, normal anal tone, brown stool

CRS CONDITION

- Rectodigital examination: -
- Colonoscope report: -
- CEA: - ng/ml
- Abnormal Lab data: -
-
  - Lung

手術日期及方法

- 手術日期及方法20200709 1. Laparoscopic anterior resection with end-to-side anastomosis, endo-GIA 60/4.1mm*2, ILS 29mm, re-inforcement with silk: nil, bilateral doughnuts: intact, air leak test: done, no air leak.
  2. High ligation (-): IMA below left colic artery.
  3. S-colon:15cm
  4. Reperitonealization: nil.
  5. Take down of splenic flexure: nil.
  6. One 12Fr. Penrose drain at pelvis.

住院治療經過

- After admission, operation of laparoscopic Anterior resection + rectopexy was smoothly performed on 20200709. With adequate post-op pain control and improving appetite. Flatus passage also noted. Also, penrose drain was removed on 7/11. Under relatively stable condition, the patient is allowed to be discharged with OPD follow up .

合併症

- nil

一般檢查

- 累積報告 :

DATE	TIME	ALB	TP	BIL.T	BIL.D	ALKP	AST	ALT	LDH	CHOL	TG	HDL-C	LDL	UA	NH3	AMY
1090708	1655	4.7		2.0	0.4	67	26	26	214							
1090713	0906			1.18	0.25											

DATE	TIME	WBC	RBC	HGB	HCT	MCV	PLT	BLST	NEUT	BAND	SEG	LYM	MON	EOS	BAS
1090708	1615	7080	4.94	15.0	44.7	90.5	199		60.0			32.3	5.5	1.8	0.4
1090713	0850	5290	4.08	12.2	37.5	91.9	164		57.1			31.2	7.9	3.2	0.6

DATE	TIME	NA	K	CL	CA	BUN	CREAT	Mg	P	CRP	ESR	PCT	LIPASE	LACTATE
1090708	1655	138	4.3	102	9.6	13	1.33							
1090713	0906	139	4.0			9	1.01							

DATE	TIME	GLUCOSE	PAB	TRF	ZN	CU	KETONE	CK	CKMB	PT-P	PT-C	APTT-P	APTT-C	TRO-T
1090708	1615									11.0	10.3	30.3		
1090708	1652	101												
1090709	0906	76												

- 放射線報告 :**檢驗項目 :CHEST PA VIEW** 報告日期 **:1090710**
  主　訴：
  Result:
  Finding:
  > The bony structure seems to be intact.
  > Normal heart size.
  > Mildly Increased interstitial infiltration over both lungs
  Impression:
  As above description.

特殊檢查

病理報告

其它

出院時情況

- 3.改門診治療

出院指示

-

藥名	劑量	劑量單位	用法	頻次	數量	數量單位	現況	天數	單位
Ultracet tab	1	TAB	PO	Q6HPRN	12	TAB	TH	3	天

出院計劃

- 門診日期
    - date on 2020/07/29
- 門診追蹤

主治醫師 2062F 蔣鋒帆 / 住院醫師 9889F 陳俊男

正式摘要(電子簽章：000000196551781)

W92 / W92

doctorRank:B
